# High-Intensity Use of Smartphone Can Significantly Increase the Diagnostic Rate and Severity of Dry Eye

**DOI:** 10.3389/fmed.2022.829271

**Published:** 2022-04-26

**Authors:** Chunyang Wang, Kelan Yuan, Yujie Mou, Yaying Wu, Xin Wang, Renjian Hu, Jinjin Min, Xiaodan Huang, Xiuming Jin

**Affiliations:** Eye Center, The Second Affiliated Hospital, School of Medicine, Zhejiang University, Hangzhou, China

**Keywords:** dry eye, smartphone, ocular surface, dry eye diagnosis, dry eye severity

## Abstract

**Purpose:**

To investigate the effects of high-intensity use of smartphones on ocular surface homeostasis and to explore whether high-intensity use of handheld digital devices can cause false increase of dry eye diagnostic rate.

**Methods:**

In this prospective self-control study, 60 subjects (120 eyes) were recruited and asked to read on smartphones provided by the same manufacturer for two consecutive hours. This study was conducted during 8:00 – 10:00 AM to eliminate the influence of digital equipment used the previous day. Ophthalmological examinations [non-invasive tear breakup time (NIBUT), fluorescein breakup time (FBUT), Schirmer I test, corneal fluorescein staining (CFS), bulbar conjunctival redness and meibomian gland (MG) assessment] and a questionnaire survey were conducted before and after the reading test. Based on the collected data, the changes in ocular surface damage and subjective symptoms of the subjects were evaluated, and the differences in the diagnostic rate of dry eye before and after high-intensity use of smartphones were compared.

**Results:**

The diagnostic rate of dry eye was sharply increased (61.7% vs. 74.2%). The severity of dry eye also changed significantly, and the moderate and severe degree increased after reading (10% vs. 15%; 5% vs. 10.8%). The aggravated severity subjects had lower MG expressibility and more evident bulbar conjunctival redness compared to the non-aggravated severity subjects. After 2 h of continuous reading, NIBUT-First, NIBUT-Average and FBUT-Average were significantly decreased, while the proportion of BUT ≤ 5 s increased significantly. Non-invasive keratograph tear meniscus height(NIKTMH) decreased significantly compared to the baseline level, while the proportion of NIKTMH<0.20 mm increased significantly. No significant difference was observed in the Schirmer I test and CFS score between the two groups. Compared to the baseline, evident aggravation was observed in bulbar conjunctival redness. The Ocular Surface Disease Index (OSDI) was significantly higher than the baseline after the reading test.

**Conclusion:**

Diagnostic indicators related to dry eye are rapidly deteriorating after high-intensity smartphone use, especially those with lower MG expressibility and ocular redness. High-intensity smartphone use can increase the false positive rate of dry eye diagnosis by disturbing ocular surface homeostasis.

## Introduction

Recently, the incidence rate of dry eye has been further increasing. On one hand, this is related to the evident increase in clinical diagnosis and treatment level, and on the other hand, to the change in modern lifestyle. Considerable evidence has revealed that dry eye is a lifestyle disease (1, 2). Dry eye can be induced by many unhealthy lifestyle chioces, including overuse of digital devices, lack of sleep and physical activity, and prolonged sedentary behaviors (3). Thus, the diagnosis of dry eye is affected by many behavioral factors, of which the overuse of handheld digital devices is a significant factor. Digital eye strain (DES) is defined as the visual disturbance and ocular discomfort associated with the use of digital devices (4). The prevalence of DES-induced symptoms is estimated to be between 25 and 93% (4–8). Dry eye is considered a major contributor to DES, and its prevalence is significantly higher among frequent visual display terminal (VDT) users. Several studies conducted in Japan have shown that VDT use is a potential risk factor for dry eye (3, 9, 10). A cross-sectional Caucasian study conducted in Italy also proved that an increase in the number of VDT workers is accompanied by an increase in the frequency of occurrence of dry eye (11).

The rapid increase in the prevalence of dry eye is putting a heavy health and economic burden on modern people. Mild-to-moderate dry eye has become a common health problem affecting people’s quality of life and work efficiency, while severe dry eye can lead to visual impairment, and even blindness, which is a complex ocular surface and corneal disease that is difficult to treat at present. Therefore, we wonder whether there is a problem of overdiagnosis in such a high diagnostic rate of dry eye, and whether a lifestyle change in a short time can cause a change in the diagnostic rate of dry eye?

Our results indicated that high-intensity smartphone use significantly interfered with the homeostasis of the ocular surface, especially the stability of the tear film. Moreover, high-intensity smartphone use sharply increased the diagnostic rate and severity of dry eye of the same group of subjects in a short period, which can cause some confusion in dry eye diagnosis. Perhaps the high-intensity use of Smartphone or tablet screen can be used as a provocation test for some patients who are at the critical threshold for the diagnosis of dry eye. Therefore, in the actual clinical work, we need to completely analyze the actual situation of patients, clarify the importance of carefully inquiring the history, and avoid the false increase in the diagnostic rate of dry eye for non-dry eye persons due to the temporary overuse of electronic equipment.

## Materials and Methods

### Subjects and Experimental Design

We conducted a prospective study at the Eye Center of The Second Affiliated Hospital of Zhejiang University School of Medicine. This study was conducted in conformance with the ethical principles of the Declaration of Helsinki and was approved by the Ethics Committee of The Second Affiliated Hospital of Zhejiang University School of Medicine. A total of 60 subjects (120 eyes) were involved in this study. The inclusion criteria were volunteers over 18 years old who used smartphones frequently and had basic reading comprehension skills. The exclusion criteria were volunteers who took any eyedrops or other treatments within 1 week, had ocular diseases except dry eye, used contact lenses within 1 month, or had undergone eye surgery within 6 months. Breastfeeding or pregnant women and people with severe systemic diseases, psychosis or dementia were also excluded. Informed consent was obtained from all subjects prior to the participating.

The subjects were asked to read for two consecutive hours on smartphones provided by the same manufacturer (Hisense Communications Co., Ltd., Qingdao, China) under sufficient ambient brightness conditions from 8:00 to 10:00 AM. The settings of all smartphones were kept the same: the screen brightness of the screens was 50% of the maximum brightness, the same app was used for reading, and the font and size of the text were the same. The distance between the eyes and the smartphone screen was fixed at 40 cm. Both before and after the reading test, the subjects underwent ophthalmological examinations and were administered questionnaires. We evaluated their dry eye-related symptoms and signs and compared the differences in the dry eye diagnostic rates before and after the reading test.

### The Diagnostic Criteria for Dry Eye

The diagnostic criteria for dry eye refer to experts’ consensus about clinical diagnosis and treatment of dry eye, published by Corneal Disease Group of Ophthalmological Society, Chinese Medical Association in 2020. They are listed as follows. (1) One of the subjective symptoms of dryness—foreign body sensation, burning sensation, fatigue, discomfort, vision fluctuation (Ocular Surface Disease Index (OSDI) ≥ 13), and non-invasive tear breakup time (NIBUT) ≤ 5 s or Schirmer I test (without surface anesthesia) ≤ 5 mm/5 min—can diagnose dry eye (2) One of the subjective symptoms of dryness, foreign body sensation, burning sensation, fatigue, discomfort, vision fluctuation (OSDI ≥ 13) and 5 s < NIBUT ≤ 10 s or 5 mm/5 min < Schirmer I test results (without surface anesthesia) ≤ 10 mm/5 min and positive corneal fluorescein staining (CFS ≥ 1) can be used to diagnose dry eye. The severity criteria for dry eye refer to experts’ consensus about clinical diagnosis and treatment of dry eye published by the Corneal Disease Group of Ophthalmological Society, Chinese Medical Association in 2020. They are listed as follows. (1) Mild: under a slit lamp microscope, there were no evident signs of ocular surface injury (corneal fluorescein staining ≤ 5), and BUT ≥ 2 s. (2) Moderate: under the slit lamp microscope, the range of corneal injury was no more than two quadrants or corneal fluorescein staining points > 5 and < 30, and BUT ≥ 2 s. (3) Severe:under the slit lamp microscope, the range of corneal injury was more than two quadrants (including two quadrants) or corneal fluorescent staining points ≥ 30, and BUT < 2 s. The fluorescein staining spots of the cornea fused into coarse spots or flakes or were accompanied by filaments. If the result of the Schirmer test is 0, it indicates a severe dry eye.

### Subjective Ocular Symptoms and Asthenopia Assessment

The subjective ocular symptoms and asthenopia were assessed using the Ocular Surface Disease Index (OSDI). which quantifies dry eye symptoms based on three subscales: ocular symptoms (OSDI Symptom), visual tasks (OSDI Visual Function) and environmental triggers (OSDI Triggers) (12). The dry eye screening criteria for the OSDI questionnaire according to the TFOS DEWS II Diagnostic Methodology report are listed as follows: Mild = 13–22, Moderate = 23–32, and Severe ≥ 33 (13).

### Ophthalmological Examination

#### Non-invasive Tear Breakup Time, Non-invasive Keratograph Tear Meniscus Height, and Bulbar Conjunctival Redness

The Keratograph 5M (Oculus, Wetzlar, Germany) was used to measure the non-invasive tear breakup time (NIBUT) and bulbar conjunctival redness. NIBUT-First was recorded as the time (in seconds) between the last complete blink and the first appearance of discontinuity in the Placido disk. NIBUT-Average was defined as the average time of BUT points during the entire examination period. The non-invasive keratograph tear meniscus height (NIKTMH) was measured as the distance between the edge of the tear meniscus and eyelid margin on the vertical line through the pupil midpoint. Ocular redness including nasal and temporal bulbar conjunctival redness, was also measured using the Keratograph 5M. The measurement was repeated for three times, with intervals of 5 min, and the average value was taken.

#### Fluorescein Breakup Time and Corneal Fluorescein Staining

The fluorescein breakup time (FBUT) measurement and vital corneal staining were performed using fluorescein sodium strips purchased Liaoning Meizilin Pharmaceutical Co., Ltd., Shenyang, China. FBUT was recorded as the time interval between the last complete blink and the appearance of the first dry spot on the tear film surface under cobalt blue light. The measurement was repeated for three times and the average value (FBUT Average) was taken.

Two minutes after the FBUT measurement, corneal fluorescein staining (CFS) was evaluated using the National Eye Institute grading system. Briefly, each cornea was divided into five corneal zones, and each corneal zone was scored from 0 to 3. The scored as follows:0 = no evident signs of ocular surface injury, 1 = corneal fluorescein staining points ≥1 and <30, 2 = corneal fluorescein staining points >30 without fused points, 3 = corneal fluorescein staining points fused into coarse spots or flakes or accompanied by filaments. The final CFS score was the sum of the five zones ranging from 0 to 15.

#### Schirmer I Test

The schirmer I test without topical anesthesia was performed using filter paper strips (Liaoning Meizilin Pharmaceutical Co., Ltd., Shenyang, China) after completing all other examinations. To avoid any influence of CFS on the schirmer I test results, the test interval was kept at least 15 min.

#### Meibomian Gland Assessment

Fifteen meibomian glands (MGs) in the lower eyelid (including five MGs each on the nasal, medial, and temporal sides) were used to assess the expressibility of MGs and quality of the secreta. MG expressibility was graded on a scale of 0 to 3 (0 = all glands expressible, 1 = 3–4 glands expressible, 2 = 1–2 glands expressible, and 3 = no glands expressible). The three groups of MGs scores were combined to obtain the total MG expressibility score ranging from 0 to 9. In addition, MG quality was graded on a scale of 0 to 3 (0 = lipid clear and transparent, 1 = lipid dirty, 2 = lipid dirty with scraps, and 3 = lipid thick like toothpaste). All MGs scores were combined to obtain the total MG quality score ranging from 0 to 45.

### Statistical Analysis

The comparisons of data before and after the test were conducted using the Wilcoxon matched-pairs signed rank test and Kruskal–Wallis H test. The baseline data were compared using in the independent samples T test and Wilcoxon Mann–Whitney rank test. The difference between right and left eyes were conducted using the Wilcoxon matched-pairs signed rank test and Kruskal–Wallis H test. Statistical analyses were conducted using a GraphPad Prism 7.0 (GraphPad Software Inc., San Diego, CA, United States). The differences were considered statistically significant when the *p*-value was less than 0.05.

## Results

### Baseline Characteristics of Subjects

A total of 60 subjects (120 eyes), including 21 males and 39 females, were involved in this study. Their mean age was 24.82 years, ranging from 22 to 30 years old. No statistically significant difference was detected between right and left eyes for related signs of dry eye disease. The baseline characteristics of subjects’ ophthalmological examinations and questionnaires are shown in Table 1.

**TABLE 1:** Baseline characteristics of subjects.

Characteristics	
Age	24.82 ± 1.84
Sex (male/female)	60 (21/39)
Smartphone usage time(year)	8.22 ± 2.31
NIBUT-first (s)	6.29 ± 2.19
NIBUT-average (s)	7.72 ± 1.91
FBUT-average (s)	5.44 ± 3.33
CFS	0.29 ± 0.67
Schirmer I test (mm)	13.45 ± 10.65
Nasal redness	0.82 ± 0.32
Temporal redness	0.82 ± 0.29
MG expressibility	1.14 ± 1.49
OSDI total	16.62 ± 11.01
CVS-Q total	13.98 ± 10.40
DEQS total	19.40 ± 11.29

*Data are expressed as the mean ± standard deviation.*

*NIBUT, non-invasive break-up time; FBUT, fluorescein break-up time; OSDI, ocular surface disease index; CVS-Q, computer vision syndrome questionnaire; DEQS, dry eye-related quality-of-life score.*

### High-Intensity Use of Smartphones Can Significantly Increase the Diagnostic Rate of Dry Eye and the Severity of Dry Eyes

**FIGURE 1:**
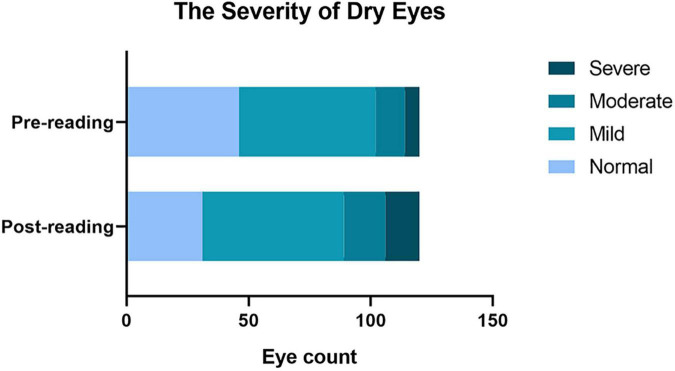
Effects of continuous reading for 2 h with smartphones in the diagnostic rate of dry eye and the severity of dry eyes. A significant increase in the total diagnostic rate of dry eye. The diagnosis rate from mild dry eye to moderate and severe eye was significantly increased compared to that before the reading test.

First, we compared the difference in the diagnostic rate of dry eye before and after the reading test and found that high-intensity smartphone use caused a sharp increase in the diagnostic rate within a short period of time (Table 2, 61.67% vs. 74.17%, *p* = 0.01). In addition, the severity of dry eye changed significantly after the reading test (Figure 1), and the moderate and severe degrees increased (Table 2, 10% vs. 15%; 5% vs. 10.8%).

**TABLE 2:** Changes of the diagnostic rate.

	The diagnostic rate						
	Normal eye	Mild dry eye	Moderate dry eye	Severe dry eye	Total number of dry eye	*Z*	*P*
Pre-reading(%)	46(38.3%)	56(46.7%)	12(10.0%)	6(5.0%)	74(61.7%)	−2.568	0.01
Post-reading(%)	31(25.8%)	58(48.4%)	18(15.0%)	13(10.8%)	89(74.2%)		

*Data are expressed as the number and percentage of eyes in different severity.*

### Increase in the Severity of Dry Eyes Caused by the High-Intensity Use of Mobile Phones Was Related to Meibomian Gland Expressibility and Ocular Redness

**FIGURE 2:**
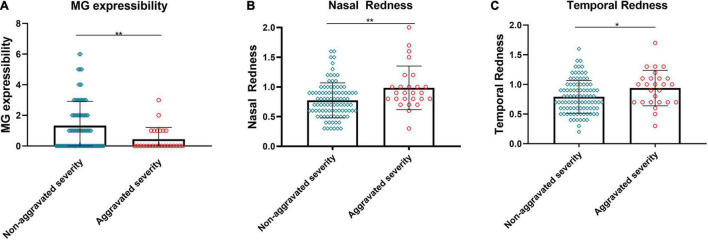
Relationship between changes in severity and baseline MG expressibility and ocular redness. The MG expressibility **(A)** of aggravated severity subjects before the reading test was significantly lower than those of non-aggravated severity subjects. Evident aggravation was observed in nasal **(B)** and temporal **(C)** bulbar conjunctival redness for subjects with aggravated severity. **(A–C)** Dates are presented as median and interquartile range, *p ≤ 0.05, **p ≤ 0.01.

Certain subjects experienced an aggravation severity after the reading test. These subjects had lower MG expressibility before the test compared to the subjects without significant changes (Figure 2A, *p* < 0.01). The redness of the bulbar conjunctiva on the nasal and temporal sides was more evident (Figure 2B, *p* < 0.01; Figure 2C, *p* < 0.05).

### Risk Factors for Patients With Increased Severity of Dry Eye

Taking the aggravation of dry eye diagnosis as the dependent variable, the independent variables were analyzed by single factor analysis, the relevant variables were screened, and then a logistic regression analysis was performed. The results showed that MG discharge and the use time of mobile phones were the protective factors causing the aggravation of dry eye diagnosis, while nasal red eye was the risk factor (Table 3, *p* < 0.05).

**TABLE 3:** Multivariate logistic analysis of the increase in the severity of dry eyes.

Characteristics	B	Walds	*P*	OR	95%CI
Smartphone usage time	−0.285	5.435	0.020	0.752	0.592–0.956
Nasal redness	1.765	5.253	0.022	5.841	1.291–26.419
MG expressibility	−0.85	6.666	0.010	0.428	0.224–0.815
					

### High-Intensity Use of Smartphone Resulted in a Significant Decrease in Tear Film Stability

**FIGURE 3:**
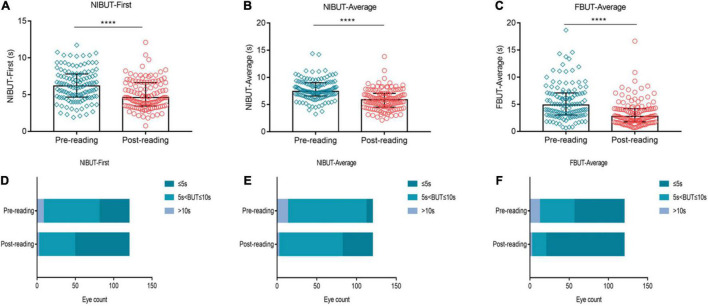
Effects of continuous smartphne reading for 2 h on tear film stability. NIBUT-First **(A)**, NIBUT-Average **(B)** and FBUT-Average **(C)** decreased significantly compared to the baseline level, while the proportion of BUT ≤ 5 s increased significantly **(D–F)**. **(A–C)** Dates are presented as median and interquartile range, ****p ≤ 0.0001.

We assessed the effects of high-intensity use of smartphones on the stability of the tear film. The results showed that, compared to the pre-reading group, NIBUT-First significantly decreased after 2 h of continuous reading (Figure 3A, *p* ≤ 0.0001), while the proportion of NIBUT-First ≤ 5 s increased significantly (Figure 3D, 32.5% vs. 59.17%). Similarly, NIBUT-Average (Figure 3B, *p* ≤ 0.0001) and FBUT-Average (Figure 3C, *p* ≤ 0.0001) also decreased significantly from the baseline level, while the proportion of BUT ≤ 5 s increased significantly (Figures 3E,F, 6.67% vs. 31.67%, 53.33% vs. 83.33%, respectively).

### Effects of High-Intensity Use of Smartphone on Tear Volume

**FIGURE 4:**
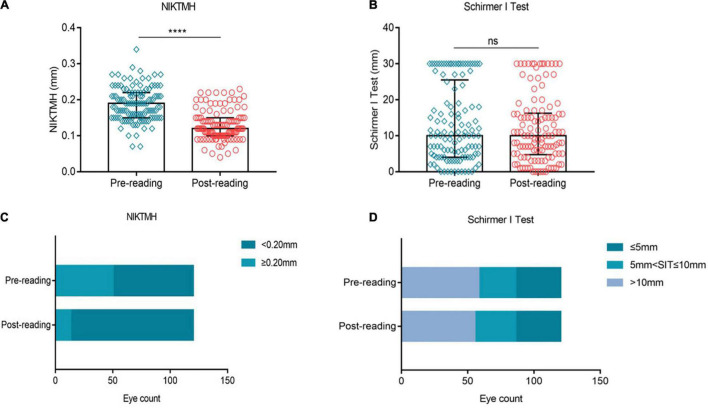
Effects of continuous smartphones reading for 2 h on tear volume. Compared to the baseline level, NIKTMH **(A)** decreased significantly, while the proportion of NIKTMH < 0.20 mm increased significantly **(C)**. The Schirmer I test **(B)** and the proportion of the Schirmer I test ≤ 5 mm **(D)** did not change significantly between the two groups. **(A–B)** Dates are presented as median and interquartile range, ****p ≤ 0.0001.

We investigated the effects of high-intensity smartphone use on tear secretion. Our results showed that compared to the baseline level, NIKTMH was significantly decreased after reading (Figure 4A, *p* ≤ 0.0001), while the proportion of NIKTMH < 0.20 mm increased significantly (Figure 4C, 58.33% vs. 89.17%). No significant difference was observed in the Schirmer I test between the two groups (Figure 4B, *P* > 0.05), and the proportion of Schirmer I test ≤ 5 mm did not change significantly before and after the reading test (Figure 4D, 28.33% vs. 28.33%).

### Excessive Use of Smartphone Aggravated Ocular Redness

**FIGURE 5:**
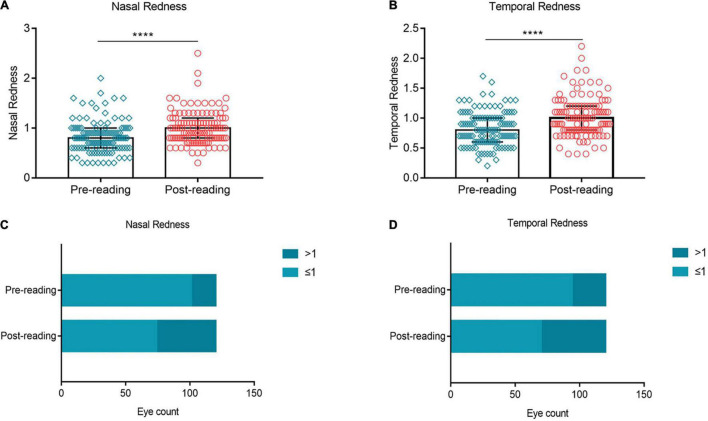
Effects of continuous smartphone reading for 2 h on ocular redness. Evident aggravation was observed in nasal **(A,C)** and temporal **(B,D)** bulbar conjunctival redness after the reading test. **(A–B)** Dates are presented as median and interquartile range, ****p ≤ 0.0001.

In this study, ocular redness was evaluated by measuring nasal and temporal bulbar conjunctival redness. Compared to the baseline, evident aggravation was observed in the nasal bulbar conjunctival redness after the reading test (Figure 5A, *p* ≤ 0.0001). The proportion of nasal bulbar redness index (BRI) > 1 grade was increased from 15.83% at baseline to 38.33% after the reading test (Figure 5C). At the same time, the temporal bulbar conjunctival redness (Figure 5B, *p* ≤ 0.0001) and the proportion of temporal BRI > 1 grade (Figure 5D, 21.67% vs. 41.67%) showed a similar trend of change trend.

### Corneal Fluorescein Staining, Meibomian Gland Expressibility, and Meibomian Gland Quality Did Not Change Significantly Over the Task

**FIGURE 6:**
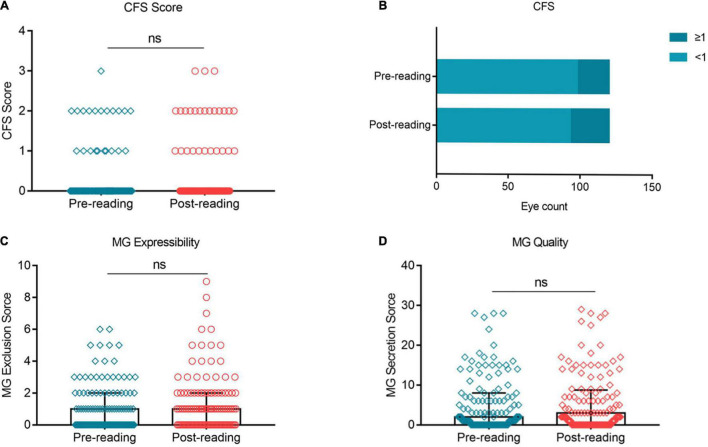
Effects of continuous smartphone reading for 2 h on CFS, MG expressibility and MG quality. No significant difference was observed in CFS **(A,B)**, MG expressibility **(C)** and MG quality **(D)** between the two groups. **(A–C)** Dates are presented as median and interquartile range.

No significant change was observed in the CFS score (Figure 6A, *p* > 0.05) and the proportion of CFS ≥ 1 between the two groups (Figure 6B, 18.33% vs. 22.5%). Similarly, the MG expressibility (Figure 6C, *p* > 0.05) and MG quality (Figure 6D, *p* > 0.05) did not change significantly after the reading test.

### High-Intensity Use of Smartphone Exacerbated Subjective Symptoms Significantly

**FIGURE 7:**
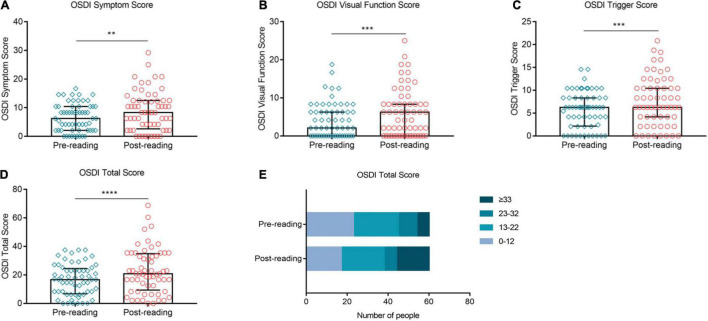
Effects of continuous smartphone reading for 2 h on OSDI, CVS-Q and DEQS. OSDI Symptom score **(A)**, visual function score **(B)**, triggers score **(C)** and total score **(D)** were increased significantly compared to the baseline level. The proportion of OSDI ≤ 12 decreased significantly, while that of OSDI ≥ 33 increased distinctively during the task **(E)**. **(A–D)** Dates are presented as median and interquartile range, **p ≤ 0.01, ***p ≤ 0.001, ****p ≤ 0.0001.

Figure 7 showed the impact of high-intensity smartphone use on subjective symptoms. The OSDI Symptom score, OSDI Visual Function score and OSDI Triggers score were significantly higher than the baseline after the reading test (Figures 7A–C, *p* ≤ 0.01, *p* ≤ 0.001, and *p* ≤ 0.001, respectively). The overall OSDI score was also significantly higher than the baseline (Figure 7D, *p* ≤ 0.0001). Furthermore, the proportion of OSDI ≤ 12 decreased from 38.33% at baseline to 28.33% after continuous reading for 2 h (Figure 7E). In contrary, the proportion of OSD ≥ 33 increased distinctively from 10% at baseline to 26.67% after the reading test (Figure 7E).

## Discussion

After using the mobile phone screen for continuous reading for 2 h, besides the misdiagnosis of non-dry eyes, the dry eye severity of certain subjects changed from mild dry eyes to moderate dry eyes, or moderate dry eyes to severe dry eyes. The dry eye test index of these people showed a more unstable trend. Our data showed that these patients had lower baseline MG expressibility and more evident nasal and temporal eye redness. Kyei et al. showed that the correlation between symptoms and signs of dry eye is low and inconsistent (14). Therefore, our severity classification criteria were dominated by corneal damage and BUT. As stated above, conjunctiva redness is closely related to ocular surface inflammation. Studies have shown that overexposure to short-wave blue light induces oxidative damage and apoptosis of the cornea, which can manifest as increased ocular surface inflammation, leading to dry eyes (15). One possible reason for this is that the subjects with severe bulbar conjunctival redness are more sensitive to the blue light emitted from smartphones and already have inflammation and damage on the eye surface before the reading test. Continuous blue light stimulation from smart phones can aggravate dry eye signs and increase the severity of diagnosis. In addition, the function of meibomian gland also influences the classification of dry eye severity. Studies have shown significantly worse fluorescein scores, tear film rupture time and Schirmer test scores in water-deficient DED patients compared with meibomian gland dysfunction (MGD), and that increased tear production in MGD can compensate for tear film rupture time (16, 17). We believe that patients with severe dry eye are more likely to have dry eye with water deficiency; thus the MG function is better. As meibomian abnormalities are chronic and diffuse, the symptoms and signs might not change much in the short term. In addition, the later the age of starting mobile phone use, the shorter is the number of years of using mobile phones, symptoms and signs of dry eye are milder and it can also be proved that the use of smart phones does have an adverse impact on dry eye diagnosis. A meta-analysis showed that the prevalence of DED increased with continued VDT use, especially with daily over 8 h, which is consistent with our findings (18).

We know that age is a risk factor associated with dry eye. Our study focused on the younger age group (23–30 years old) and found that for younger age groups, the prevalence of dry eye disease is also gradually increasing due to long-term TVD use and the effects of prolonged screen time during the COVID-19 pandemic. Barabino S et al. found that compared with older patients, younger people had more severe subjective symptoms, thinner lipid thickness but relatively normal meibomian gland function (19). This may also explain why the evaporative type with the greatest impact in this study, while there was no significant change in meibomian gland function. J. Murube et al. proposed different classification methods for dry eye (20). The classification of etiology points out that with the increase of age, the exocrine glands of the human body, including the lacrimal gland, will undergo progressive apoptosis, and it is easy to form dry eye due to insufficient tear secretion, resulting in an increase in the incidence and severity of dry eye (20). Because the body adjustment ability of the elderly is worse than that of the young, and the results of tear film break-up time and schirmer test caused by dehydration DED are more serious, the changes in physical signs of the results of this study are also applicable to the elderly. In addition, studies have shown that Asians were at a higher risk of DED than Caucasians, so this study is of great significance for the exploration of the incidence of dry eye in China (21). However, due to an insufficient number of sample, we did not find an association between gender and the dry eye diagnosis rate of short-term intensive use of mobile phone screens.

At present, dry eye diagnosis is mainly based on subjective symptoms, tear function abnormalities and positive vital staining. However, we found that dry eye related indicators, such as BUT and NIKTMH can change significantly in the short term after external intervention. For example, the proportion of subjects with NIBUT ≤ 5 s increased rapidly from 32.5% at baseline to 59.17% after continuous reading on a smartphone for 2 h. The reason for the dramatic change in BUT during the reading test is attributable to the decreased blink rate, incomplete blinks, increased ocular surface exposure, and increased tear evaporation caused by excessive smartphone use (22). Moreover, the change trend of the indicators reflecting the tear volume was not consistent, which was reflected in the fact that the proportion of subjects with NIKTMH ≤ 0.20 mm increased rapidly after reading, while, the Schirmer I test results did not change significantly during the test. The reason for this difference is attributable to the different detection methods of the two indicators. In this study, the Schirmer I test was performed without topical anesthesia, so it would have induced a heavy stimulation on the ocular surface, which can even mask the influence of excessive smartphone use on the tear volume (23). Compared to the Schirmer I test, NIKTMH is a non-invasive examination, which can detect the change in tear volume in a more microscopic manner and is less affected by the operation (24). No significant difference was found before and after reading with fluorescence staining. We believe that the ocular surface injury is less likely to change in the short term. Because the diagnostic criteria of dry eye are directly related to OSDI, NIBUT, Schirmer I test and positive corneal fluorescein staining, the abnormal tear function of patients in a short period can lead to aggravation of symptoms, which is finally reflected in the increase in the diagnosis rate.

The new definition of dry eye created by TFOS DEWS II emphasizes the important role of ocular surface inflammation in dry eye development. Conjunctival redness is the most common clinical sign suggesting ocular surface inflammation (25, 26). According to our study results, high-intensity smartphone use resulted in a significant increase in bulbar conjunctival redness. The high-intensity blue light (450–470 nm) emitted from smartphones is considered to be a risk factor for ocular surface inflammation. Marek et al. reported that blue light was phototoxic to human conjunctival and corneal epithelial cells. It induces cell death, stimulates ROS formation, and changes the expression of inflammatory genes. They found that it was much worse under hyperosmotic condition, which suggested that patients with dry eye are more sensitive to blue light (27). In addition, the magnetic field (MF) released from smartphone is another important factor in eye surface damage. Keklikci et al. suggested that low frequency MF exposure leads the morphological changes in the conjunctiva and decreased the quantity of goblet cells (28). Other studies have shown that MF can also cause ocular surface damage by promoting the apoptosis of epithelial cells, increasing oxidative stress, and promoting the expression of inflammatory genes (29–31). All of these factors may be responsible for the increase in the subjects’ ocular surface redness index. According to our study results, dry eye-related indicators, such as BUT, NIKTMH, and ocular redness index, are prone to fluctuations when disturbed by unhealthy lifestyle habits, which lead to a false increase in the diagnostic rate of dry eye.

In addition, it is well known that the accommodation of the eye, including dry eye symptoms and signs, has a circadian rhythm. García N et al. also confirmed that the clearance of fluorescein sodium from tears was lower at night than during the day (32). There are also studies showing that the subjective symptoms of eye discomfort and eye redness in patients with dry eye are milder during the day than at night (33). All of these studies showed an impact on the diagnosis of dry eye at different time periods, but the course of the study included at least two longer intervals, such as two hours each at morning and evening or at noon and evening. However, some studies have also shown that although tear meniscus height in patients with dry eye has a downward trend within one day (detected every 3 h), there is no statistically significant difference (34). Rodriguez et al. found a time node at 12:00 noon about ocular discomfort and corneal staining, but there was also no statistical difference (35). We think that there is no significant difference in the symptoms and signs of dry eye in a short period of time (within 2 h), and the subjective symptoms and objective signs in the morning are mild, so we arrange the examination time of each subject at 8:00 in the morning. Therefore, the influence of different times of one day on the rhythm of the tear apparatus, meibomian glands, tear film stability and corneal inflammation was excluded, and the possible influence of the use of electronic devices on participants before the start of the experiment was also avoided as much as possible.

## Conclusion

In conclusion, high-intensity use of smartphone results in a significant decrease in tear film stability, an evident aggravation in ocular redness and ocular discomfort symptom, leading to a sharp increase in the diagnostic rate and severity of dry eye in a short period of time. As a lifestyle disease, the diagnostic indicators of dry eye can be easily disturbed by unhealthy lifestyle habits. In the current diagnosis and treatment process, due to the large number of people in the outpatient department, patients generally need to wait in line for more than dozens of minutes before they can see a doctor. This time interval increases the probability of patients using video terminals such as smart phones before the examination. Moreover, dry eye patients with severe ocular inflammation but better meibomian gland function are more likely to be misdiagnosed and aggravated. Alternatively, high-intensity smartphone or tablet screen use may serve as a provocative test for some patients at critical thresholds for diagnosing dry eye. Therefore, we need to analyze the actual situation of patients fully in the actual clinical work and avoid the false increase in the diagnostic rate of dry eye due to the temporary overuse of electronic equipment. A limitation of this study is that it does not analyze how the influence of continuous reading screen disappears, which requires further exploration in the future.

## Data Availability Statement

The original contributions presented in the study are included in the article/Supplementary Material, further inquiries can be directed to the corresponding author.

## Ethics Statement

The studies involving human participants were reviewed and approved by the Human Body Research Ethics Committee of the Second Affiliated Hospital of Zhejiang University. The patients/participants provided their written informed consent to participate in this study.

## Author Contributions

XJ designed the study and reviewed and edited the manuscript. CW, KY, YW, RH, XW, JM, and XH conducted the test. KY and YM collected the data and did the statistical analysis. CW and KY wrote the first draft of the report. All authors contributed to manuscript revision, read, and approved the submitted version.

## Conflict of Interest

The authors declare that the research was conducted in the absence of any commercial or financial relationships that could be construed as a potential conflict of interest.

## Publisher’s Note

All claims expressed in this article are solely those of the authors and do not necessarily represent those of their affiliated organizations, or those of the publisher, the editors and the reviewers. Any product that may be evaluated in this article, or claim that may be made by its manufacturer, is not guaranteed or endorsed by the publisher.
